# Proteomic Profiling of Neuroblastoma Cells Adhesion on Hyaluronic Acid-Based Surface for Neural Tissue Engineering

**DOI:** 10.1155/2016/1917394

**Published:** 2016-12-07

**Authors:** Ming-Hui Yang, Ko-Chin Chen, Pei-Wen Chiang, Tze-Wen Chung, Wan-Jou Chen, Pei-Yu Chu, Sharon Chia-Ju Chen, Yi-Shan Lu, Cheng-Hui Yuan, Ming-Chen Wang, Chia-Yang Lin, Ying-Fong Huang, Shiang-Bin Jong, Po-Chiao Lin, Yu-Chang Tyan

**Affiliations:** ^1^Center for Infectious Disease and Cancer Research, Kaohsiung Medical University, Kaohsiung 807, Taiwan; ^2^Department of Pathology, Changhua Christian Hospital, Changhua 500, Taiwan; ^3^Department of Medical Imaging and Radiological Sciences, Kaohsiung Medical University, Kaohsiung 807, Taiwan; ^4^Department of Biomedical Engineering, National Yang-Ming University, Taipei 112, Taiwan; ^5^Department of Laboratory Medicine, Kaohsiung Medical University Hospital, Kaohsiung 807, Taiwan; ^6^Department of Medical Laboratory Science and Biotechnology, College of Health Sciences, Kaohsiung Medical University, Kaohsiung 807, Taiwan; ^7^Office of Safety, Health and Environment, Kaohsiung Medical University, Kaohsiung 807, Taiwan; ^8^Mass Spectrometry Laboratory, Chemical, Molecular and Materials Analysis Center, Department of Chemistry, National University of Singapore, Singapore 119077; ^9^Department of Biomedical Engineering, Chung Yuan Christian University, Chungli 300, Taiwan; ^10^Department of Nuclear Medicine, Kaohsiung Medical University Hospital, Kaohsiung 807, Taiwan; ^11^Department of Chemistry, National Sun Yat-sen University, Kaohsiung 804, Taiwan; ^12^Graduate Institute of Medicine, College of Medicine, Kaohsiung Medical University, Kaohsiung 807, Taiwan; ^13^Institute of Medical Science and Technology, National Sun Yat-sen University, Kaohsiung 804, Taiwan

## Abstract

The microenvironment of neuron cells plays a crucial role in regulating neural development and regeneration. Hyaluronic acid (HA) biomaterial has been applied in a wide range of medical and biological fields and plays important roles in neural regeneration. PC12 cells have been reported to be capable of endogenous NGF synthesis and secretion. The purpose of this research was to assess the effect of HA biomaterial combining with PC12 cells conditioned media (PC12 CM) in neural regeneration. Using SH-SY5Y cells as an experimental model, we found that supporting with PC12 CM enhanced HA function in SH-SY5Y cell proliferation and adhesion. Through RP-nano-UPLC-ESI-MS/MS analyses, we identified increased expression of HSP60 and RanBP2 in SH-SY5Y cells grown on HA-modified surface with cotreatment of PC12 CM. Moreover, we also identified factors that were secreted from PC12 cells and may promote SH-SY5Y cell proliferation and adhesion. Here, we proposed a biomaterial surface enriched with neurotrophic factors for nerve regeneration application.

## 1. Introduction

Nerve injury is an important topic in the world of medicine and there are many nerve injury cases reported every year. These injuries usually have caused a decreased quality of life because of reduction in motor, sensory and autonomic functions [1, 2]. The peripheral nervous system is more permissive to axonal regeneration then the central nervous system; but it is still a challenge to surgery [3]. Despite microsurgical techniques which are more advanced, experimental and clinical evidences show the results of peripheral nerve recovery are not satisfying [4, 5]. Nerve autograft is the gold standard technique for therapy in peripheral nerve injury. A tubular nerve guidance channel is necessary for nerve autograft. It acts as a physical guide for nerve regeneration and provides a conduit for neurotrophic factor diffusion from the injured nerve stumps [3, 6, 7]. In the past few years, scientists have focused on various conduit materials, including aliphatic polyesters [8, 9], poly(phosphoesters) [10], polyurethanes [11], piezoelectric polymers [12], hydrogel-based nerve guide channels [13], collagen [14], polysaccharides [15], and decellularized biomatrices [3, 16].

Hyaluronan (hyaluronic acid, HA), a component of the extracellular matrix, is a glycosaminoglycan applicable to biomaterial. During embryogenesis, the concentration of HA is at the peak in undifferentiated cells and decreases at the beginning of cell differentiation [17]. Such change is crucial for the angiogenic process regulation and its presence in the extracellular matrix (ECM) is as a naturally occurring polysaccharide [18–20]. HA is vital in the brain development, especially to the postnatal brain in regions adjacent to the lateral ventricles where stem cells reside [21, 22]. It has been reported as a significant factor in a wide range of medical and biological fields, such as reactive oxygen species, angiogenesis, cancer, lung injury, liver injury, kidney injury, brain injury, diabetes, and leukocyte trafficking and in immune regulation [23, 24]. It also plays important roles in neural proliferation, differentiation, migration, survival, and cell signaling [25]. HA-induced signal transduction depends on the interactions of cell surface receptors, including cluster determinant 44 (CD44) and toll-like receptor 4 (TLR4) [26]. In the central nervous system, the HA expression level is elevated at damaged sites. The high molecular weight HA has been digested through hyaluronidases becoming smaller fragments; such products activate downstream signal transduction to regulate progenitor cell differentiation and proliferation to promote nerve repair [25]. It also has advantages as a scaffold material and can be combined with adhesive peptides or other ECM components to provide cell attachment. Previous study indicated that combination scaffolds consisting of fibrin with HA and laminin provide biomaterial properties to enable polymerization with cells. This mimics the native tissue of the brain and supports differentiation of human neural stem/progenitor cell (hNSPC) function [27]. Currently, HA-based biomaterials are used to regulate the cell differentiation and studied for tissue engineering purposes in combination with growth factors or ECM components for tissue repair [28–33].

Cell to cell interaction is important for cell fate determination, providing the first evidence for short-range regulatory mechanisms of cell differentiation. The conditioned medium (CM), which contains growth factors and differentiation regulation factors that are released from the cultured cells, could be used to promote cell differentiation into specific lineages [34]. Previous reports indicated that mouse embryonic stem cells (mESCs) treated with HepG2 CM can enhance mesoderm induction and the subsequent osteogenic differentiation of mESCs [35]. In addition, human marrow stromal cells (hMSC) CM can stimulate the induction of the mesodermal lineage and subsequent differentiation toward the osteogenic and chondrogenic lineage [36]. Differentiation of the human umbilical cord blood neuronal progenitors (HUCBNPs) was achieved by treatment with human SH-SY5Y CM, which showed an increase of the ratio of long outgrowths to cell body diameter and a characteristic of developing neurons [37]. PC12 cells require no supplementary NGF for survival and proliferation because they synthesize and secrete endogenous NGF into the medium as an autocrine regulation, which may be applied for neuron development [38]. The combination of PC12 CM and HA surface biomaterials may synergistically induce the neuronal cell differentiation, offering a new field of vision in nerve regeneration.

In this study, we examined the effects of HA and PC12 CM in SH-SY5Y cells. SH-SY5Y is one kind of human derived cell line which is used in scientific research. The original cell line, called SK-N-SH, was subcloned and isolated from a bone marrow biopsy, which had been taken from a female with neuroblastoma. This cell line has been widely used as a model of neuron diseases as these cells possess many biochemical and functional properties of neurons. SH-SY5Y cells have been widely used as in vitro models of neurological studies, including analysis of neuronal differentiation, metabolism, and function related to neurodegenerative and neuroadaptive processes, neurotoxicity, and neuroprotection. It can be differentiated to a more mature neuron-like phenotype that is characterized by dopaminergic markers and, as such, has been used to study Parkinson's disease [39]. Through the investigation, proteins that influence the responses and later proliferation of SH-SY5Y cells on HA-biopolymer surfaces and PC12 CM were identified. Using proteomic approaches to assess characteristic proteins from HA and PC12 CM treatment found that heat shock protein 60 (HSP60) and E3 SUMO-protein ligase RanBP2 were involved in cell proliferation and attachment regulated the UBC/PI3K/AKT1/mTOR pathway.

## 2. Materials and Methods

### 2.1. HA Surfaces Determined by QCM Measurements

The surface of a 9 MHz QCM gold electrode (ANT Tech, Taiwan) was washed with 1 M HCl, rinsed with DI water, followed by drying at room temperature. The frequency of the electrode measured by the QCM (ADS, ANT Tech, Taiwan) was assigned as *F*_0_ and the flow rate was 60 *μ*L/min of phosphate buffered saline (PBS). To prepare QCM-HA layers, HA was adsorbed onto a QCM electrode surface using the layer-by-layer technique. The HA solution (0.5%, Lifecore Biomedical, Inc., USA) was injected into the flow loop of the QCM electrode at flow rate of 60 mL/min and the frequency shifts of the QCM were measured. The frequency shifts determined by the QCM were recorded and the mass of HA adsorption was calculated. To test whether HA layer is stably coated onto the electrode, the frequency of the electrode was measured by the flow of PBS for several minutes. For tested HA-biopolymer, the frequency shift dropped sharply, it was absorbed onto the electrode surface. The detection theory for QCM can be explained by the Sauerbrey equation (1), which calculates that the mass change is proportional to the oscillation frequency shift of the piezoelectric quartz crystal.

(1) $$\begin{array}{c} \Delta F=-2.3\times 10^{-6}\frac{F^{2}\Delta M}{A} \end{array}$$

In Sauerbrey equation (1) in gas phase, Δ*F* is the frequency shift (Hz); *F* is basic oscillation frequency of piezoelectric quartz (Hz); *A* is the active area of QCM (cm^2^); Δ*M* is the mass change on QCM (g).

### 2.2. Culturing SH-SY5Y Cells on the Electrodes Decorated by HA Surfaces

For seeding SH-SY5Y cells, the HA-modified electrodes, decorated by biopolymer layers, were sterilized with 70% (v/v) ethanol and then exposed to ultraviolet light. The 4 × 10^4^ SH-SY5Y cells in serum-free medium were added to each well in the presence of the aforementioned electrodes and incubated at 37°C in 5% CO_2_ for 12 hours for investigation of the adhesion of the cells on those electrodes. After the incubations, the electrodes were washed with PBS, and then frequency shifts were measured by the QCM to quantify the adhesions of SH-SY5Y cells on electrodes.

### 2.3. HA Surface Characterized by FT-IR

The surface characterization of the coverslips decorated with HA was observed using a Fourier transform infrared spectrometer (FT-IR, Spectrum One system, PerkinElmer, USA).

### 2.4. Cell Culture and Conditioned Media (CM) Collection

For human neuroblastoma cell line, SH-SY5Y cells were cultured in Dulbecco's Modified Eagle Medium: nutrient mixture F-12 (DMEM/F12) medium (Gibco, Invitrogen, USA) with 10% FBS plus 1% antibiotics. In the pheochromocytoma cell line of the rat adrenal medulla, PC12 cells were maintained in DMEM medium with 10% horse serum, 5% FBS plus 1% antibiotics. Those cells were incubated in 5% CO_2_ at 37°C for 48 hours.

In this study, PC12 CM were collected, filtered, and mixed with equal volumes of fresh DMEM/F12 medium for SH-SY5Y cells to be cultivated on HA surface. To collect PC12 CM, the PC12 cells were rinsed with phosphate buffer saline (PBS) and then incubated in serum-free DMEM medium for 12 hours. Then, the supernatants of the medium were collected and filtered with 0.22 *μ*m filter.

### 2.5. Protein Preparation for Proteomic Analysis

SH-SY5Y cell lysates or PC12 CM were transferred into 1.5 mL tubes and reduced with 1 M dithiothreitol (DTT, USB Corporation, USA) in 25 mM NH_4_HCO_3_ at 37°C. After 3 hours, protein samples were alkylated with 1 M iodoacetamide (IAA, Amersham Biosciences, USA) in the dark at room temperature for 30 min. After the proteins were digested by sequencing-grade modified porcine trypsin (Promega, USA) overnight at 37°C, 2 *μ*L of formic acid was added to each sample.

RP-nano-UPLC ESI-MS/MS analyses (nanoACQUITY UPLC, Waters, Milford, MA, coupled to an ion trap mass spectrometer, LTQ Orbitrap Discovery Hybrid FTMS, Thermo, San Jose, CA) were conducted according to standard procedures described below. Briefly, a sample of the desired peptide digest was loaded into the reverse phase column (symmetry C18, 5 *μ*m, and 180 *μ*m × 20 mm). The RP separation was performed using a linear acetonitrile gradient from 99% buffer A (100% DI water/0.1% formic acid) to 85% buffer B (100% acetonitrile/0.1% formic acid) in 120 min using the micropump at a flow rate of approximately 400 nL/min. The separation was performed on a C18 microcapillary column (BEH C18, 1.7 *μ*m, and 75 *μ*m × 100 mm). As peptides were eluted from the microcapillary column, they were electrosprayed into the ESI-MS/MS with the application of a distal 2.1 kV spraying voltage with heated capillary temperature of 200°C. Each cycle of one full-scan mass spectrum (*m*/*z* 400–2000) was followed by three data dependent tandem mass spectra with collision energy set at 35% [40].

All MS and MS/MS data were analyzed and processed using the Mascot software (Version 2.2.1, Matrix Science, London, UK) against the Swiss-Prot database. The search parameters were set as follows: 0.5 Da for MS/MS tolerance, 10 ppm for MS tolerance, carbamidomethylation (C) as the fixed modification, deamidated (NQ), oxidation (M), phospho (ST) and phospho (Y) as the variable modification, and 2 for missing cleavage. Proteins were initially annotated by similar search conditions using UniProtKB/Swiss-Prot databases (SIB Swiss Institute of Bioinformatics, Lausanne, Switzerland). The protein-protein interaction pathways were performed by String 9.1 Web software (SIB Swiss Institute of Bioinformatics, Lausanne, Switzerland) [41].

### 2.6. Western Blotting of Protein Expression

Confirmation of protein identities was performed by Western blotting. Protein extracts were prepared in lysis buffer and each cell lysate sample (1 *μ*g/*μ*L, 10 *μ*L) was electrophoresed through a precast gel (NuPAGE®Novex® 4–12% Bis-Tris Gel, 1.5 mm, 10 wells, Invitrogen™, Carlsbad, CA). Proteins were transferred from the gel to a polyvinyldifluoride (PVDF) membrane (Millipore, Bedford, CA) by means of the semidry technique using the Criterion Blotter (Bio-Rad) at 100 V for 60 min and blocked with 5% milk in PBS (adjusted to pH 7.4) containing 0.05% Tween-20. The membranes were then separately incubated overnight with primary rabbit antibodies (1 *μ*g/*μ*L). The commercially available primary antibodies used in this study included the following: monoclonal mouse anti-HSP60 (Stressgen, USA) and polyclonal rabbit anti-RanBP2 (Abcam, USA). After washing, the membrane was incubated with HRP-conjugated goat anti-mouse IgG antibodies (purchased from Jackson ImmunoResearch, USA) for 1 hour (1 : 10000). Proteins were detected with an enhanced chemiluminescent (ECL) system, and quantitative analysis of Western blotting was carried out using the ImageQuant-TL-7.0 software, version 2010 (Amersham Biosciences).

### 2.7. BrdU Assay

The cell viability was determined by BrdU cell proliferation assay kit (Millipore). The assay was performed according to the manufacturer's instructions. Briefly, 1 × 10^3^ SH-SY5Y cells were seeded in a sterile 96-well tissue culture plate and incubated for 24 to 72 hours. Then, cells were incubated in the culture medium containing BrdU reagent for 2 hours. Fixing solution was added before the absorbance was measured at 450 nm using an ELISA reader (Multiskan EX, Thermo Scientific, Vantaa, Finland).

### 2.8. Cell Morphology Observed by Immunofluorescence Staining

The SH-SY5Y cells were grown on coverslips in 12-well culture plates. After 24 hours' incubation, the cells were fixed (60% methanol and 40% acetone) at −20°C for 30 min and then permeabilized (0.5% Triton X-100) at room temperature for 5 min. After rinsing with PBS, the cells were blocked (6% bovine serum albumin) and then incubated with primary and secondary antibodies. The nuclei and cytoskeleton of the cells were stained with DAPI (Sigma-Aldrich, USA), vimentin (Vimentin DyLight 488 Antibody, Epitomics, USA), monoclonal mouse anti-HSP60 (Stressgen, USA), and polyclonal rabbit anti-RanBP2 (Abcam, USA), respectively. After rinsing with PBS, the cells were mounted with ProLong® Gold Antifade Reagent (Invitrogen). The images were acquired by a microscope equipped with fluorescence light source (FLoid Cell Fluorescence Imaging Station, Invitrogen).

### 2.9. Statistical Analysis

All calculations used the SigmaStat statistical software (Jandel Science Corp., San Rafael, CA, USA). All statistical significance was evaluated at 95% of confidence level or better. Data are presented as mean ± standard error.

## 3. Results and Discussion

### 3.1. Quantitative Analysis of Adsorbed of HA and Adhesion of SH-SY5Y Cells on Electrodes Using QCM Techniques

In our previous studies, the QCM system was applicable to the quantitative analysis of adsorption of HA and adhesion of cells on electrodes [42, 43]. The QCM frequency variation after HA-biopolymer formation was −212.47 ± 6.33 Hz; the adsorbed HA mass corresponding to those surfaces was 228.11 ± 3.30 ng. To investigate the adhesion of SH-SY5Y cells onto electrodes decorated by HA surface with PC12 CM, SH-SY5Y cells were incubated on the electrodes. The cultivation of SH-SY5Y cells under serum-free conditions after 12 hours herein prevented the apoptosis and proliferation of cells, which were then changed to DMEM/F12 medium without or with PC12 CM. The results concerning the adhesion of SH-SY5Y cells onto the electrode of QCM that was decorated by HA were obtained from the frequency shifts. The frequency shifts for HA-modified surfaces without or with PC12 CM were from −3.68 ± 0.42 to −10.47 ± 0.27 × 10^3^ Hz; the attached cell mass corresponding to HA surface was from 1.13 ± 0.13 to 3.22 ± 0.08 × 10^3^ ng, respectively (Table 1, *n* = 10). With treatment of PC12 CM after 48 and 72 hours, the frequency shift was lowered and the mass of cell was increased from that of regular medium. These results indicated that PC12 CM may be beneficial to SH-SY5Y cells adhesion or proliferation.

### 3.2. Investigation of HA Structure by FT-IR

**Figure 1:**
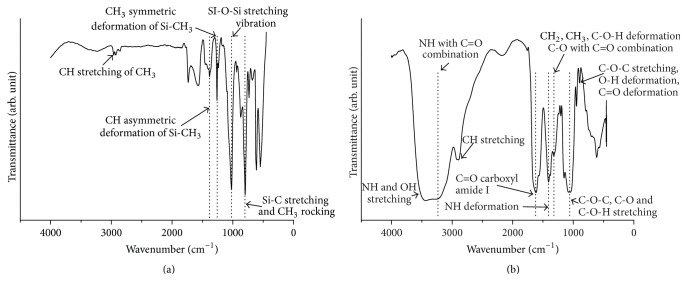
The FT-IR spectra show the frequency region from 4000 to 500 cm^−1^ of modified surfaces and (a) nonmodified and (b) HA-modified coverslips.

HA-modified surfaces of coverslips were also routinely characterized using FT-IR spectra. The FR-IR spectra in the range of 500–4000 cm^−1^ for HA surfaces were presented in Figure 1. Expansion of the FT-IR spectra in Figure 1 clearly showed the difference between spectra of nonmodified and HA-modified coverslips. The HA-modified surface showed several sharp peaks such as at 894.9 and 1049.1 cm^−1^ that could be due to the C-O-C stretching, at 1321.0 cm^−1^ that corresponds to the presence of C-O with C=O combination, at 1406.9 cm^−1^ that indicates the presence of NH deformation, at 1616.1 cm^−1^ due to the C=O carboxyl amide I, and at 2893.8 to 3433.8 cm^−1^ that confirms the presence of CH stretching, and NH with C=O combination and OH stretching. Similar peaks were indicated in Table 2. These peaks obtained in the HA-modified surface share a highly similar position when compared to the standard HA.

### 3.3. Combination Treatment of HA and PC12 CM Increased SH-SY5Y Cell Proliferation

**Figure 2:**
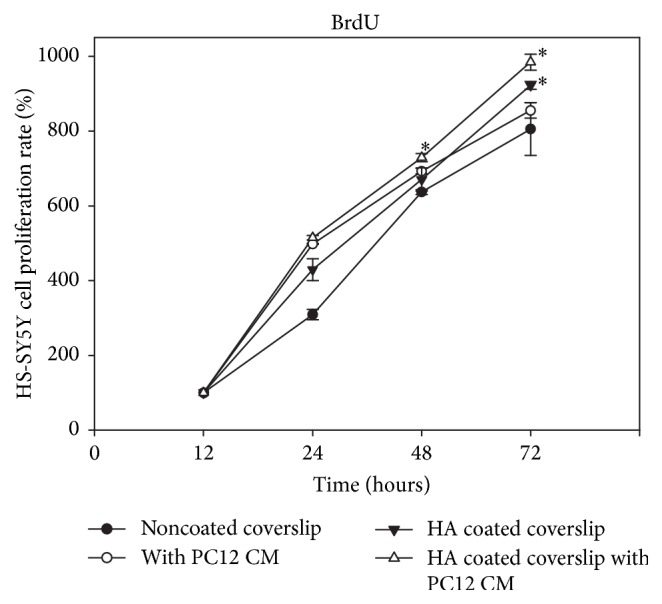
The relative percentage of cell viability obtained from BrdU cell proliferation assay. Differential treatments of SH-SY5Y cells were seeded in 96-well tissue culture plates and incubated for 24 to 72 h. Cells were treated with BrdU reagent for 2 h and fixed before the absorbance was measured at 450 nm.

HA is well known to promote fibroblasts proliferation and enhance cell adhesion [42]. To investigate the HA and PC12 CM effect on SH-SY5Y cells, the cell proliferation was assessed. The 4 × 10^4^ SH-SY5Y cells were seeded and grown on HA-modified and nonmodified coverslips. The cell proliferation was measured by BrdU assay for 24, 48, or 72 hours and the baseline of cell proliferation was set at 12 hours after seeding. As shown in Figure 2, SH-SY5Y cell proliferation rates on HA-modified coverslips were similar to those of nonmodified coverslips of 48 hours. However, the SH-SY5Y cell proliferation rate was increased significantly after 72 hours on the HA-modified coverslips.

The PC12 CM was collected and added to the culture medium of the SH-SY5Y cell. After 48 hours' incubation, the results showed that PC12 CM induced SH-SY5Y cell growth and proliferation especially with the HA-modified surface. The HA-modified coverslip combined with treatment of PC12 CM promoted SH-SY5Y cell proliferation after 72 hours' incubation as indicated in Figure 2 using BrdU assay. Therefore, HA and PC12 CM were the two factors with a synergistic effect.

To understand the mechanism and consequence of the increasing of the SH-SY5Y cell proliferation and growth, the proteins in PC12 CM and SH-SY5Y cell lysate were identified by proteomic approaches.

### 3.4. Identification of Regulator Secreted from PC12

To identify the PC12 secreted proteins related to SH-SY5Y cell proliferation and cell adhesion, the original PC12 CM were collected and the proteins were identified by RP-nano-HPLC-ESI-MS/MS. One hundred seventy-three HA-modified surface proteins were identified and then narrowed down to the number 62, using a threshold of a minimum of three peptides identified in a protein. We found that several proteins (described below) are involved in cell differentiation functions.  Table 3 shows the details of the protein identification (protein accession number, protein name, biological process, and molecular function) in PC12 CM.

The expression of Gametogenetin (GGN) was confined to late pachytene spermatocytes and round spermatids, a time window concomitant with the occurrence of meiosis. It was expressed with highest level in diplotene spermatocytes and meiotic germ cells, especially when the nuclear membrane breaks down and the nucleolus is disorganized. In addition to functioning in proliferation of primordial germ cells, POG also involved in spermatogenesis [44].

Adrenomedullin (ADM) is a member of the calcitonin gene-related peptide (CGRP) family, which has shown neuroprotective functions [45]. ADM is secreted in many organs and tissues [46], and so were PC12 cells. ADM mediates downstream signaling through calcitonin receptor-like receptor (CRL)-receptor-activity-modifying proteins (RAMPs) complex [46].

Spermatid perinuclear RNA-binding protein (SPNR) is a microtubule-associated RNA-binding protein [47]. SPNR gene has been detected in the testis, ovary, and brain [48]. Mice deficient for SPNR show neurologic, spermatogenic, and sperm morphological abnormalities [49]. In our study, SPNR was detected in PC12 CM. This finding indicates that SPNR may be involved in neuron cell development.

The Pro-neuregulin-2 (Nrg2) has played a critical role in the growth and development of multiple organ systems, which was also involved in neural and organ development. In the embryo, the Nrg2 was expressed in the brain where it was found in the telencephalon, but not in the hindbrain. The Nrg2 was direct ligand for ErbB 3 and ErbB 4 tyrosine kinase receptors. Concomitantly recruiting ErbB 1 and ErbB 2 coreceptors, the Nrg2 may result in ligand-stimulated tyrosine phosphorylation and activation of the ErbB receptors, which may also promote the heterodimerization with the EGF receptor [50].

NRG1/ErbB signaling pathways are important in CNS development and may be neuroprotective in brain injury [51]. ErbB4 is predominantly expressed in the brain and well characterized for its function in the CNS [52]. In the CNS, NRG1/ErbB4 signaling is involved in neuronal migration, dendritic spine maturation, and the formation of inhibitory synapses onto excitatory pyramidal neurons [51]. ErbB4 mutant mice were showed to alter the organization and migration of neuroblast chain and display olfactory interneurons deficits in the placement and differentiation [53].

The complement system plays an important role in inflammatory diseases and neurodegenerative processes of the CNS [54]. C3a, one of complement factors, has been shown to be involved in synaptic refinement regulation and neuronal survival during development in the CNS [55].

The interaction of Notch, with its established intercellular signaling pathway, plays a key role in neural development. The Notch-3 activation induces the increase of the progenitor cell number in the central nervous system (CNS) and affects CNS development [56]. The Notch-3 mutation may lead to cerebral autosomal dominant arteriopathy with subcortical infarcts and leukoencephalopathy (CADASIL). CADASIL leads to stroke and dementia and is the main feature of recurrent subcortical ischemic events and vascular dementia. Members of the Notch gene family were thought to be involved as receptors for membrane-bound ligands Jagged1, Jagged2, and Delta1 in the regulation of cell fate in a variety of neurogeneses of embryos, particularly in the developing CNS from the homogenous cell population of the neural tube [57, 58].

**Figure 3:**
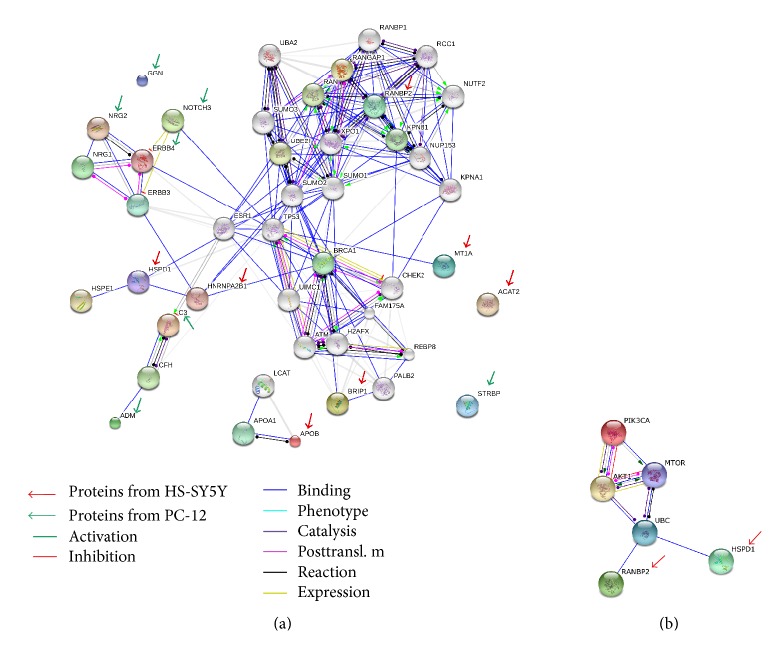
The protein-protein interaction pathways are illustrated. (a) Proteins identified in this study are marked by arrows (red: SH-SY5Y; green: PC12). (b) Two proteins, HSP60 and RanBP2, may turn on the ubiquitin (UBC) pathway, which is responsible for the proliferation and is required for survival of the majority of cells.

Also, in this study, more than one hundred proteins were identified in SH-SY5Y cell lysate and most of these were identified at the minimal confidence level, which was only one unique peptide sequence matched. Experimental results reported a total of six protein identifications with higher confidence levels (at least three unique peptide sequences matched). The protein-protein interaction pathways were performed by String 9.1 Web software, and proteins identified in this study were marked by arrows (red: SH-SY5Y; green: PC12; Figure 3(a)). Using the protein-protein interaction pathway analysis, the main finding of PC12 CM-treated cells is that the growth factors may focus on the enhancement of the TP53 pathway in SH-SY5Y cells which may result in cell growth (Figure 3(b)).

The TP53 pathway has been famously recognized to be connected to the UBC/PI3K/AKT1/mTOR pathway, which is responsible for the proliferation and is required for survival of the majority of cells. The hypothesis of the mTOR pathway is that it acts as a master switch of cellular catabolism and anabolism, thereby determining whether cells grow and proliferate. In particular, the UBC/PI3K/AKT1/mTOR pathway regulates the import and retention of glucose. It provides substrates for glycolysis and the biosynthetic pathways which rely on the supply of glycolytic intermediates. The mTOR pathway, downstream of AKT signaling, regulates the protein translation rate and accelerates the supply of amino acid biosynthesis to generate the charged tRNAs [59].

To confirm this hypothesis, the proteins in SH-SY5Y cell lysate need to be validated. In addition, there were two proteins, 60 kDa heat shock protein (HSP60, known as HSPD1) and E3 SUMO-protein ligase RanBP2 (RanBP2), identified in SH-SY5Y cell lysate samples, which were involved in cell proliferation, differentiation, development, and cycle regulation. Those two proteins were also involved in the UBC/PI3K/AKT1/mTOR pathway (Figure 3(b)). To corroborate the protein candidates identified by RP-nano-HPLC-ESI-MS/MS, the Western blot analysis and immunofluorescence staining were applied to detect the changes in HSP60 and RanBP2.

**Figure 4:**
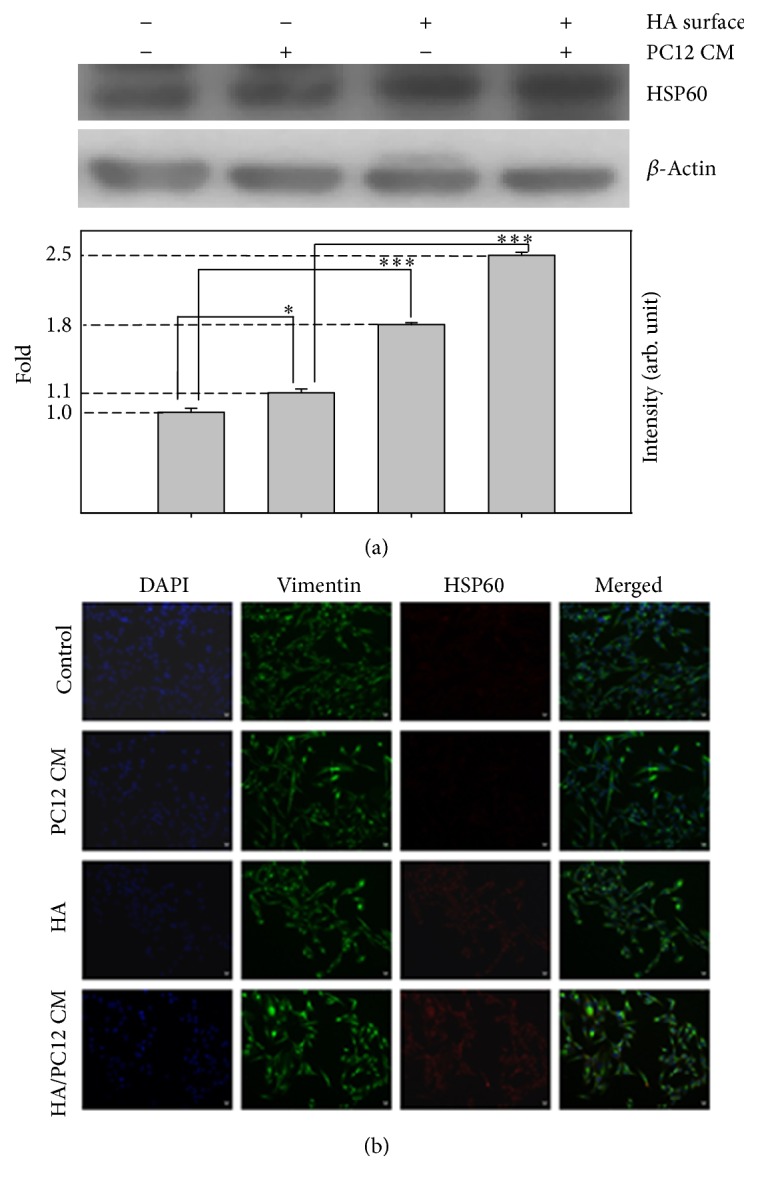
The detection of HSP60 protein expression on SH-SY5Y cells. (a) Western blotting of HSP60 and *β*-actin from SH-SY5Y cells cultured on different surfaces and/or different medium. The signals were quantified and the data are presented as the means ± SEMs; *p* < 0.05 or 0.001 indicates statistical significance, as determined by unpaired Student's *t*-test. (b) Analyses of representative samples of SH-SY5Y cells expression of vimentin and HSP60 are shown. Immunochemical stains for DAPI (blue), vimentin (green), and HSP60 (red) for adhered SH-SY5Y cells on indicated surfaces and/or conditioned media for 24 h (scale bars, 10 *μ*m; confocal microscope, 400x). ^*∗*^ *p* < 0.05 and ^*∗∗∗*^ *p* < 0.001.

Heat shock proteins (HSPs) are overexpressed in a wide range of cells and are implicated in cell proliferation, differentiation, and recognition by the immune system. These proteins have molecular chaperone activity, which can be induced by various environmental stresses. Some HSPs were found to be localized in the synapse [60]. HSP60 is widely distributed in the brain and involved in neurodegenerative disorders [61]. When injured, HSP60 can be released to activate microglia in CNS [62]. HSP60 defects can cause neurodegenerative pathologies, such as brain hypomyelination and leukodystrophy [63]. Here, we found the protein expressions of HSP60 were upregulated in HA-modified surface or PC12 CM-treated SH-SY5Y cells compared to control, and the effect was significantly enhanced by the above-mentioned combination (Figure 4(a)). Immunofluorescence staining also showed that cotreatment with HA-modified surface and PC12 CM increased HSP60 expression in SH-SY5Y cells (Figure 4(b)). These results suggested that HA and PC12 CM may trigger HSP60 as part of its nerve regeneration effects.

**Figure 5:**
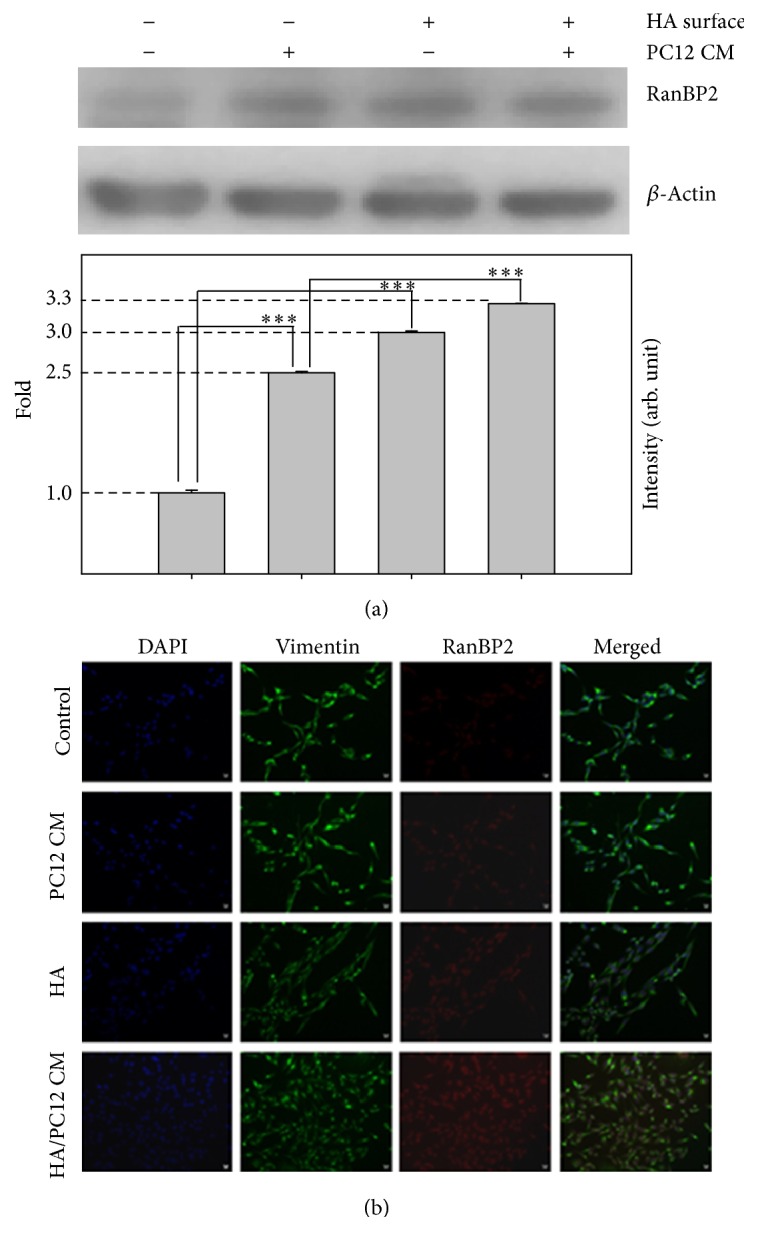
The detection of RanBP2 protein expression on SH-SY5Y cells. (a) Western blotting of RanBP2 and *β*-actin from SH-SY5Y cells cultured on different surfaces and/or different medium. The signals were quantified and the data are presented as the means ± SEMs; *p* < 0.001 indicates statistical significance, as determined by unpaired Student's *t*-test. (b) Analyses of representative samples of SH-SY5Y cells expression of vimentin and RanBP2 are shown. Immunochemical stains for DAPI (blue), vimentin (green), and RanBP2 (red) for adhered SH-SY5Y cells on indicated surfaces and/or conditioned media for 24 h (scale bars, 10 *μ*m; confocal microscope, 400x). ^*∗∗∗*^ *p* < 0.001.

RanBP2, located at the nuclear pore complexes (NPCs) [64], is known to modulate CRM1-mediated nuclear protein export [65] and associate with Ubc9 to function as a SUMO E3 ligase [66–68]. RanBP2 also plays a role in neuroprotective regulation [69–71]. RanBP2 associates with RPGRIP1 to implicate retinopathies in amacrine and 661W neurons [72]. RanBP2 mutation has been identified as a key factor in acute necrotising encephalopathy [73, 74]. Here, the protein expression levels of RanBP2 in PC12 CM-treated or HA-modified surface treated SH-SY5Y cells were higher than those from control cells. Cotreatment with HA-modified surface and PC12 CM slightly increased RanBP2 protein expression compared with HA-modified surface or PC12 CM (Figure 5(a)). Immunofluorescence staining also showed the similar results that the expression of RanBP2 was increased in PC12 CM-treated and HA-modified surface with PC12 CM-treated SH-SY5Y cells (Figure 5(b)). Thus, HA and PC12 CM may upregulate RanBP2 protein expression as part of its nerve neuroprotective effects.

In the result, it showed that HA and PC12 CM may regulate protein expression, such as HSP60 and RanBP2 to promote SH-SY5Y cell proliferation and adhesion. These results were similar to Yamada's study, which reported that the combination treatment of SHya and FGF-2 increased NHA proliferation [75]. HA biomaterial surface has been used and reported in a wide range of medical and biological applications and plays an important role in neural development [25, 28]. Due to the activation of UBC/PI3K/AKT1/mTOR, signaling through mutation of pathway components as well as through activation of upstream signaling molecules occurs in a majority of cells contributing to deregulation of proliferation, resistance to apoptosis, and changes in metabolism characteristic of transforming cells.

## 4. Conclusion

In this study, SH-SYSY cells were used as a model to examine the effects of HA and PC12 CM in neuron regeneration. We found that stimulation of a HA-modified surface with PC12 CM can promote SH-SYSY cell proliferation and adhesion; the combination of both showed synergy effects on SH-SYSY cell regeneration. Our evidences supported that neurotrophic factor proteins enhance HA function in neurogenesis. Biomaterial surface supported with neurotrophic factor proteins may be utilized in nerve autograft application. We used proteomic analysis to analyze the molecular mechanisms of HA-modified surface and PC12 CM stimuli. Among these proteins, HSP60 and RanBP2 were upregulated in SH-SY5Y cells. The UBC/PI3K/AKT1/mTOR pathway was related to the cell growth and proliferation. Future study will be of the molecular regulations and interaction networks governed by biomaterials and combined with neurotrophic factors.

## Figures and Tables

**Table 1:** Frequency shifts of QCM and weights of adhered SH-SY5Y cells on the electrodes decorated with HA-modified surface for 24 to 72 hours of cell incubation.

Cell adhesion	Δ*F* (×10^3^ Hz)	Δ*m* (×10^3^ ng)
DMEM/F12 medium		
24 hrs	−3.68 ± 0.42	1.13 ± 0.13
48 hrs	−4.70 ± 0.48	1.44 ± 0.15
72 hrs	−4.93 ± 0.69	1.51 ± 0.21
DMEM/F12 medium-PC12 CM		
24 hrs	−6.30 ± 1.35	1.94 ± 0.42^*∗*^
48 hrs	−10.83 ± 0.58	3.33 ± 0.18^*∗*^
72 hrs	−10.47 ± 0.27	3.22 ± 0.08^*∗*^

Data are expressed as mean ± standard error, *n* = 10, ^*∗*^ *p* < 0.05 (*t*-test).

**Table 2:** The assignment of FT-IR bands for HA-modified surface.

Function group	Wavenumber (cm^−1^)
C-O-C stretching, O-H deformation, C=O deformation	894.9
C-O-C, C-O, C-O-H stretching	1049.1
CH_2_, CH_3 _C-O-H deformation, C-O with C=O combination	1321.0
NH deformation	1406.9
C=O carboxyl amide I	1616.1
CH stretching	2893.8
NH with C=O combination	3261.2
NH stretching and OH stretching	3433.8

**Table 3:** Proteins identified by the higher confidence level (at least three unique peptide sequences matched) in the PC12 CM which were involved in neuron generation function.

Accession numbers	Protein name	Biological process	Molecular function
Q66HC8	Gametogenetin	Cell differentiation	
		Double-strand break repair	
		Embryo implantation	
		Spermatogenesis	
P43145	ADM	Aging	Adrenomedullin receptor binding
		Androgen metabolic process	
		Calcium ion homeostasis	
		cAMP-mediated signaling	
		Hormone secretion	
		Vasculogenesis	
		Cell proliferation	
		Apoptotic process	
Q9JKU6	Spermatid perinuclear RNA-binding protein	Cell differentiation	DNA binding
		Multicellular organismal development	RNA binding
		Spermatogenesis	
O35569	Pro-neuregulin-2, membrane-bound isoform	Epidermal growth factor receptor signaling pathway	Epidermal growth factor receptor binding
		Intracellular signal transduction	ErbB-3 class receptor binding
		Organ development	
Q62956	Receptor tyrosine-protein kinase erbB-4	Cardiac muscle tissue regeneration	ATP binding
		Cell migration	Receptor signaling protein tyrosine kinase activity
		Nervous system development	Transmembrane receptor protein tyrosine kinase activity
		Apoptotic process	
		Cell proliferation	
		Glucose import	
		Odontogenesis	
		Protein tyrosine kinase Signaling pathway	
P01026	Complement C3	Blood coagulation	C5L2 anaphylatoxin chemotactic receptor binding
		Chemotaxis	Cofactor binding
		Fatty acid metabolic process	Endopeptidase inhibitor activity
		Inflammatory response	Lipid binding
		Glucose transport	
		Triglyceride biosynthetic process	
		Response to progesterone and estrogen	
Q9R172	Neurogenic locus notch homolog protein 3	Cell differentiation	Calcium ion binding
		Multicellular organismal development	
		Notch signaling pathway	
		Regulation of transcription, DNA-templated	
		Tissue regeneration	
